# Sex differences in hip and groin injury prevalence: a systematic review and meta-analysis of 3133 team-sport athletes

**DOI:** 10.5114/biolsport.2026.158668

**Published:** 2026-01-23

**Authors:** Toni Bailén-García, Marcos Quintana-Cepedal, Omar de la Calle, María Medina-Sánchez, Irene Crespo, Hugo Olmedillas

**Affiliations:** 1 Department of Functional Biology, University of Oviedo, Oviedo, Spain; 2 Asturian Research Group in Performance, Readaptation, Training, and Health (AstuRES), Oviedo, Spain; 3 Department of Surgery, University of Oviedo, Oviedo, Spain

**Keywords:** Groin pain, Injury prevention, Prevalence, Hip pain, Female athlete

## Abstract

The aim of this meta-analysis was to compare the prevalence of hip and groin injuries (HGIs) among male and female athletes. Systematic review and meta-analysis. A systematic search for related studies published from inception to February 2025 was conducted using the following databases: Cochrane, PubMed/MEDLINE, Web of Science, Embase, SPORTDiscus, PEDro and Scopus. A total of nine clinical trials, cohort or case-control studies were included. Data recorded from each study included authors, year of publication, study design, number of participants, sport played, age, number of injured athletes, injury prevalence, and type of injury (time-loss (TL) and/or non-time-loss (NTL)). Pooled odds ratio (OR) with 95% confidence intervals (CIs) were calculated using random-effects meta-analysis. The risk of bias (RoB) was evaluated with the Quality in Prognosis Studies (QUIPS) tool and the certainty of the evidence was evaluated using the Grading of Recommendations, Assessment, Development, and Evaluations (GRADE) approach. The pooled results of 3133 athletes (1955 males and 1178 females) showed no differences in HGIs between both sexes with an OR of 1.39 (95% CI: 0.99–1.96; PI: 0.48–3.99; p = 0.06). No differences were observed for TL (OR = 1.55) and NTL (OR = 0.97) injuries. Male football players showed higher odds of suffering groin problems (OR = 2.25). This systematic review and meta-analysis reveal a similar prevalence of HGIs in both males and females. The results highlight the importance of establishing screening measures for all athletes.

## INTRODUCTION

Hip and groin injuries (HGIs) are very common in sports such as football [1], hockey [2], and rugby [3], where frequent changes of direction, kicks, jumps and sprints occur [4]. Contemporary research emphasizes the importance of documenting all complaints, including non-time-loss injuries (NTL), where athletes continue to play despite experiencing decreased performance [5–7]. This approach is critical because a significant proportion (70–90%) of HGIs do not lead to activity cessation, considering time-loss (TL) injuries exclusively underestimates the real prevalence of this problem [8, 9].

The first and second steps in the sequence of injury prevention involve defining the extent of the problem and the factors that make athletes more susceptible to injury [10]. Previous groin injury, higher level of play, reduced hip adductor strength, lower levels of sportspecific training and male sex are associated with an increased risk of sustaining HGIs [4, 11]. Regarding the latter, certain anatomical differences may explain why groin injuries are more common in males compared to females. The female pelvis is wider and the angle between the inferior pubic rami is larger, resulting in different force vectors at the origin of the proximal adductors causing less strain to the muscle insertion [12]. Additionally, males are more likely to develop CAM morphology in the femoral head [13], which could increase the risk of HGIs due to changes in hip mechanics [14]. Two qualitative reviews have examined the differences in groin injury prevalence between males and females, yielding similar results. Orchard et al. [11] postulated that males have a two-fold higher incidence of groin injuries while Ross et al. [15] concluded that groin injuries are more prevalent in males, compared to females. Differentiating between injury definition (TL and NTL) and performing quantitative synthesis are vital steps for improving the quality of the evidence on this topic. Therefore, the primary objective of the present systematic review was to identify differences in HGIs prevalence between males and females. Secondary objectives were to compare prevalence according to injury definition, sport, or clinical entity. To the authors’ knowledge, this is the first meta-analysis conducted on differences in HGIs prevalence between males and females.

## MATERIALS AND METHODS

The study was prospectively registered in PROSPERO (code: CRD42023488444) and carried out following the methods described in the Cochrane handbook and is reported using the PRISMA (Preferred Reporting Items for Systematic Reviews and Meta-Analyses) guidelines [16].

### Search strategy

**FIG. 1:**
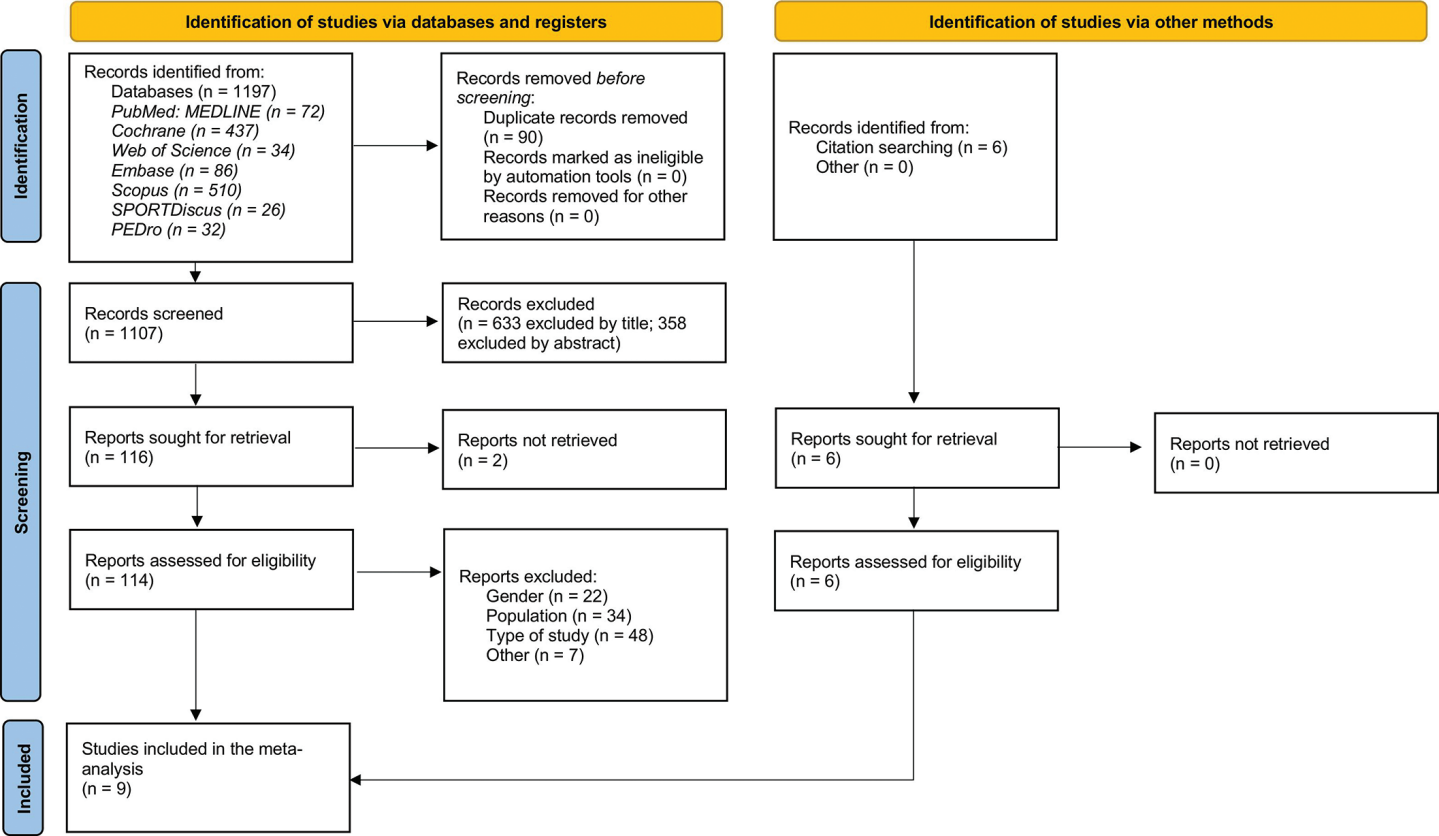
PRISMA flow chart.

A systematic search of seven databases was conducted from inception to February 2025: Cochrane, PubMed/MEDLINE, Web of Science, Embase, SPORTDiscus, PEDro and Scopus. The references of included articles were checked for further reports that did not appear in the initial search and met the inclusion criteria. Details of the search strategy can be found in Appendix 1. Two reviewers (OC and IC) independently screened the titles and abstracts and assessed the full text of all potentially eligible articles to evaluate their possible inclusion in the review. Any discrepancies were resolved during a consensus meeting, and a third reviewer was available (HO), if needed. The study selection process is presented in Figure 1.

### Study selection criteria

Eligibility criteria were established and agreed upon by all authors based on the concept of the PICOS question (population, intervention/indicator, comparator/control, outcome and study design) [16]. Thus, to be included in this systematic review and meta-analysis, studies had to fulfil the following criteria:

P) Studies had to include male and female athletes participating in competitive sports regardless of their competitive level and age.
I) The studies had to report HGIs prevalence irrespective of whether they are TL or NTL.
C) Eligible studies had to include HGIs in males and females separately.
O) Studies had to report OR or provide the necessary data for their calculation.
S) Studies were included if they were cohort, case-controls and clinical trials (control group only).

Articles had to be written in English and published in a peer-reviewed journal. ‘Grey literature’ (e.g., abstracts, conferences, theses and unpublished articles) was not considered [17]. Articles were excluded when they did not meet one inclusion criterion.

### Data collection and analysis

#### Data extraction

Data were extracted by one reviewer (MQC) and compiled in a Microsoft Excel spreadsheet. The collected data included: (1) general characteristics of the study (authors, year of publication and study design), (2) characteristics of the athletes (number of participants, sport played and age), (3) epidemiological data on injuries (number of injured athletes, injury prevalence, odds ratios (ORs) and type of injury (TL, NTL)). If necessary, the authors of the included articles were contacted to clarify possible inquiries and/or request data. No follow-up email was needed since the required data were obtained after the first contact.

#### Risk of bias assessment

The risk of bias assessment was carried out independently by two reviewers (MQC and OC) using the Quality in Prognosis Studies (QUIPS) tool. The QUIPS tool is used to evaluate the risk of bias in prognostic articles, which assess the association between a risk factor and health problems in a certain population, and has been previously described in the literature [18, 19]. The tool consists of six different areas for assessing the risk of bias: (1) study participation, (2) study attrition, (3) prognostic factor measurement, (4) outcome measurement, (5) study confounding, (6) statistical analysis and reporting. Each item is marked as ‘yes’, ‘partial’, ‘no’ or ‘unsure’. Once all items have been assessed, they are scored independently with a ‘high’, ‘moderate’ or ‘low’ risk of bias. Specific criteria to mark a section as high, moderate or low risk of bias can be found in Appendix 2. Regarding the outcome measurement domain, several studies collected hip and groin injury data through self-reported questionnaires or retrospective player reporting, with limited use of clinician-confirmed diagnoses. This characteristic was considered when applying the QUIPS criteria for rating the risk of bias in this domain.

### Quality of Evidence

The quality of the evidence (QoE) was graded using the GRADE (Grading of Recommendations Assessment Development and Evaluation) approach [20]. Initially, it was assumed that the quality was high. Quality was downgraded to moderate, low, or very low when one of the following factors was rated as serious, or very serious: risk of bias, inconsistency (i.e. high heterogeneity) [21], imprecision (i.e. wide confidence intervals) [22] or indirectness (differences in population demographics) [23].

### Statistical analysis

Raw data to calculate the effect sizes (Odds Ratios [OR]) from the studies was extracted to a Microsoft Excel sheet. Six meta-analyses were performed to explore (1) all HGIs, (2) TL injuries, (3) NTL injuries, (4) HGIs in football, (5) HGIs in hockey, and (6) injuries affecting the groin region. The usage of random effect models was decided after applying the Cochran Q test (p < 0.10), which measures between-study heterogeneity. Additionally, the variability between studies was estimated with the τ^2^ statistic. The I^2^ was used to measure the percentage of total variation in the effect sizes due to heterogeneity, with > 50% and > 75% representing moderate and high heterogeneity, respectively [24]. Forest plots were created to illustrate the effects of the different studies and the global estimation of each meta-analysis. The forest plot shows the OR of each individual study and the pooled OR for the random effect model, all with their 95% confidence intervals (CI), expressed on the logarithmic scale. Prediction intervals (PIs) were calculated to estimate the range within which the effect of a possible future study in this field might fall. The ORs were interpreted using the following benchmarks: 1.0 (trivial), 1.5 (small), 3.5 (moderate), and 9 (large) [25]. Males were considered as the exposed group (at risk). This choice was based on historical evidence suggesting higher prevalence of hip and groin injuries in male athletes compared to females, as reported in the latest systematic reviews [11, 15]. Therefore, if the calculated OR values were > 1 results indicate that males have higher odds of injury compared to females. Statistical analyses were performed using R (R version 4.3.3) with the “meta” package [26, 27].

## RESULTS

### Search results

The initial search yielded a total of 1197 articles. After removing duplicates a total of 1107 articles remained, of which 633 were excluded by title and 358 by abstract leaving a total of 116 for fulltext screening. In addition, six articles were added by other sources. Finally, nine articles met the inclusion criteria and were selected for qualitative and quantitative synthesis [7, 8, 28–34]. The flow chart showing the study selection process is presented in Figure 1.

### Description of the included studies

There were seven case-control studies [7, 8, 28–30, 32, 34], one cohort study [33], and one clinical trial [31]. Studies were published from 2014 [30] to 2024 [29] and the sports played were: football [8, 30, 31, 33], rink hockey [7, 32], ice hockey [34], gaelic games [29] and water polo [28]. Two studies were conducted in Australia [28, 33], one study was carried out in Ireland [29], one in Norway [8], three studies in Sweden [30, 31, 34], one in Spain [7], and one included an international population [32]. A total of 3133 athletes participated, of which 1955 were males (62%) and 1178 females (38%) aged 11–30, a total of 1330 athletes suffered at least one HGI (916 males and 414 females). With regard to the level of play, we found six articles featuring elite/sub-elite athletes [7, 8, 30, 32–34] and the rest of the studies collected data on amateur populations [28, 29, 31]. The duration of the studies was six weeks [8], one season [7, 28, 29, 31–34], or from career injury compilation [30]. Regarding to injury definition, there were five studies including TL injuries [7, 8, 32–34] with a total of 228 athletes injured of which 159 were males and 69 females; three studies included NTL injuries [8, 32, 33] with a total of 264 athletes injured, of which 183 were males and 81 females. In relation to sports, there were a total of 475 (76 females), 379 (140 females), 425 (166 females) and 51 (32 females) players injured in Football, Hockey, Gaelic Games and Water-Polo, respectively. There were a total of 704 players suffering injuries in the groin region [7, 8, 30, 32], of which 555 were males and 149 females. Characteristics of the included studies can be found in Table 1.

**TABLE 1:** Characteristics of included studies

Study (author, year, design, country)	Nº participants; sport, age (mean)	Nº of injuries	Type of injury: TL, NTL, other.	Type of diagnosis (self-reported, medical or other. physiotherapist) and method	Outcome (OR, 95% CI)	Risk of bias (QUIPS)
Girdwood *et al.,* 2021 Case-Control Australia	Water Polo 153 athletes (Males = 65 Females = 88) Age: 23 (all sample)	51 injuries (Males = 19 Females = 32)	None	Self-reported Online Google questionnaire and OSTRC overuse injury questionnaire	0.72 OR (0.36–1.44 95% CI)	High

Harøy *et al.,* 2017 Case-Control Norway	Football 240 athletes (Males = 195 Females = 45) Age: 23 (elite females); 24 (elite males)	132 injuries (Males = 112 Females = 20)	TL n = 42 (Males = 38 Females = 4) NTL n = 90 (Males = 74 Females = 16)	Self-reported and physiotherapist review (only in 6 of 15 teams) OSTRC overuse injury questionnaire	1.69 OR (0.88–3.24 95% CI)	Moderate

Jordan *et al.,* 2024 Case-Control Ireland	Gaelic Games 775 athletes (Males = 438 Females = 337) Age: 22.18 (all sample)	425 injuries (Males = 259 Females = 166)	None	Self-reported In-Person/Online survey via Survey Monkey	1.49 OR (1.12–1.98 95% CI)	Low

Karlsson *et al.,* 2014 Case-Control Sweden	Football 623 athletes (Males = 479 Females = 144) Age: 22.7 (males with groin injury); 21.3 (females with groin injury); 22.3 (males without groin injury); 20.7 (females without groin injury)	305 injuries (Males = 264 Females = 41)	None	Self-reported Email questionnaire	3.08 OR (2.06–4.62 95% CI)	High

Lindblom *et al.,* 2023 Two-armed cluster-randomized trial with an additional comparison arm Sweden	Football 180 athletes (Males = 62 Females = 118) Age: 20.5 (comparison group)	15 injuries (Males = 5 Females = 10)	None	Self-reported and physiotherapist review via telephone OSTRC overuse injury questionnaire	0.95 OR (0.31–2.90 95% CI)	High

Quintana-Cepedal *et al.,* 2022 Case-Control Spain	Rink Hockey 251 athletes (Males = 183 Females = 68) Age: 20 (female); 21 (male)	98 injuries (Males = 74 Females = 24)	TL n = 52 (Males = 46 Females = 6)	Self-reported Online Google Forms questionnaire	1.24 OR (0.70–2.22 95% CI)	Low

Quintana-Cepedal *et al.,* 2024 Case-Control Spain, France, Portugal	Rink Hockey 446 athletes (Males = 285 Females = 161) Age: 18 (females); 18 (males)	169 injuries (Males = 105 Females = 64)	TL n = 68 (Males = 42 Females = 26) NTL n = 169 (Males = 105 Females = 64)	Self-reported Online Google Forms questionnaire	0.88 OR (0.59–1.31 95% CI)	Low

Schoffl *et al.,* 2021 Cohort Australia	Football 105 athletes (Males = 58 Females = 47) Age: 12.9 (females); 12.5 (males)	23 injuries (Males = 18 Females = 5)	TL n = 18 (Males = 14 Females = 4) NTL n = 5 (Males = 4 Females = 1)	Physiotherapist Injury evaluation by physiotherapist and information was recorded on excel	3.78 OR (1.28–11.15 95% CI)	Moderate

Wörner *et al.,* 2023 Case-Control Sweden	Ice Hockey 360 athletes (Males = 190 Females = 170) Age: 23.2 (females); 25.3 (males)	112 injuries (Males = 60 Females = 52)	Substantial* n = 48 (Males = 19 Females = 29)	Self-reported Online survey and OSTRC overuse injury questionnaire (modified version)	1.05 OR (0.67–1.64 95% CI)	High

TL: Time-loss; NTL: Non-time-loss; QUIPS: Quality in prognosis studies; OR: Odds ratio; CI: Confidence interval; OSTRC: Oslo Sports Trauma Research Centre.

* Substantial injuries were included in TL injuries meta-analysis

### Risk of Bias

**FIG. 2:**
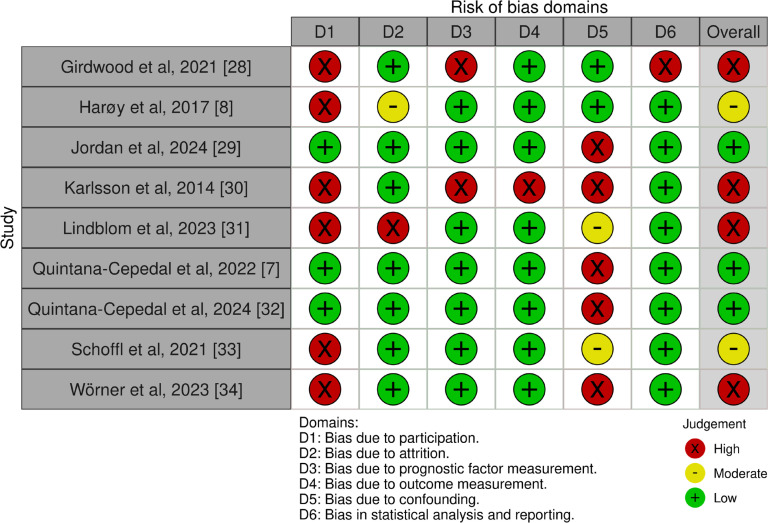
Risk of bias assessment.

Three studies (33%) presented low risk of bias [7, 29, 32], moderate risk of bias was identified for two studies (22%) [8, 33]; the rest of the studies were rated as of high risk of bias [28, 30, 31, 34]. The main source of bias was study participation [8, 28, 30, 31, 33, 34] (item 1) followed by study confounding variables [7, 29, 30, 32, 34] (item 5). Other sources of bias included prognostic factor measurement (item 3), study attrition (item 2), outcome measurement (item 4) and statistical analysis and reporting (item 6). Individual study scores for the QUIPS tool can be found in Figure 2.

### Meta-analyses

#### Overall Hip and Groin Injuries

**FIG. 3:**
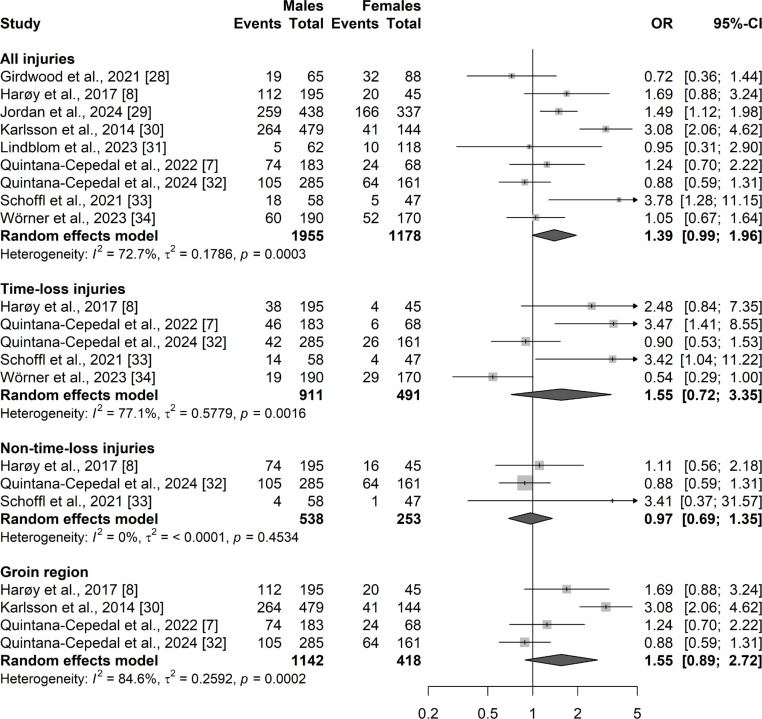
Odds ratios of injury prevalence comparison between male and female athletes. Data are sub-grouped by all injuries (HGIs), TL, NTL injuries and injuries in the groin region.

Random effects model for the overall HGIs showed a trivial effect size (OR) of 1.39 (95% CI: 0.99–1.96; PI: 0.48–3.99; *p* = 0.06; QoE: very low). Forest plots with 95% CI for all injuries can be found in Figure 3.

#### Prevalence comparison according to injury definition

Analyses were performed for both TL and NTL injuries. Random effects model for TL injuries showed a small OR of 1.55 (95% CI: 0.72–3.35; PI: 0.1–23.6; *p* = 0.26; QoE: very low). Random effects model for NTL injuries showed a trivial effect size of 0.97 (95% CI: 0.69–1.35; PI: 0.11–8.6; *p* = 0.84; QoE: high). Forest plots with 95% CI for the corresponding sub-analyses can be found in Figure 3.

#### Injuries in the groin region

Analyses were performed for studies including injuries in the groin region. Random effects model showed a small OR of 1.55 (95% CI: 0.89–2.72; PI: 0.12–19.1; *p* = 0.12; QoE: very low). Forest plots with 95% CI for injuries in the groin region can be found in Figure 3.

#### Prevalence difference by sport

**FIG. 4:**
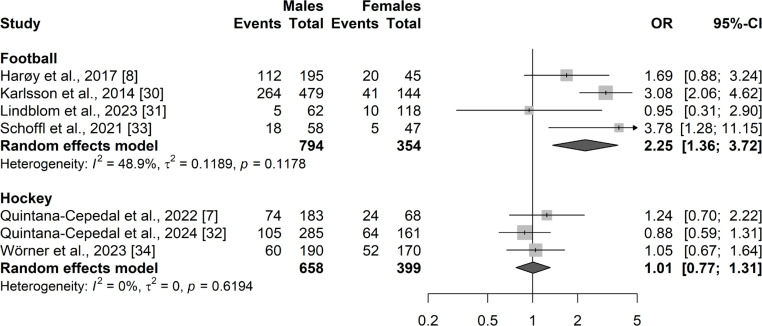
Odds ratios of injury prevalence comparison between male and female athletes. Data are sub-grouped by football and hockey.

Analyses were performed for football and hockey. Random effects model for football showed a small OR of 2.25 (95% CI: 1.36–3.72; PI: 0.35–14.3; *p* < 0.01; QoE: very low). In hockey, random effects model showed an OR of 1.01 (95% CI: 0.77–1.31; PI: 0.2–5.6; *p* = 0.96; QoE: moderate). Forest plots with 95% CI for HGIs in football and hockey can be found in Figure 4.

### Quality of evidence synthesis

The resulting quality of evidence was very low in four out of six meta-analyses, moderate quality was achieved in the analysis of hockey and high quality in the analysis of NTL injuries. The evidence profile according to the GRADE approach is presented in Table 2.

**TABLE 2:** Evidence profile according to the GRADE (Grading of Recommendations Assessment Development and Evaluation).

Outcome	Nº of studies	RoB	Inconsistency	Indirectness	Imprecision	Publication bias	Effect size	Grade score
All HGIs	9	-2	-2	0	0	NA	1.39 OR (0.99–1.96)	⊕◯◯◯ Very low

TL injuries	5	-1	-2	0	0	NA	1.55 (0.72–3.35)	⊕◯◯◯ Very low

NTL injuries	3	0	0	0	0	NA	0.97 (0.69–1.35)	⊕◯◯◯ Very low

Groin region	4	-1	-2	0	0	NA	1.55 (0.89–2.72)	⊕◯◯◯ Very low

Football	4	-2	-1	0	0	NA	2.25 (1.36–3.72)	⊕◯◯◯ Very low

Hockey	3	-1	0	0	0	NA	1.01 (0.77–1.31)	⊕◯◯◯Moderate

HGIs: Hip and Groin Injuries; RoB: Risk of Bias; NA: Not assessed (in meta-analyses of less than 10 studies.

## DISCUSSION

This is the first systematic review and meta-analysis that compared differences in HGIs prevalence between males and females. The key findings are: (1) males and females have similar overall prevalence of HGIs; (2) no differences are observed regarding TL and NTL injuries, and (3) injury prevalence differs in football players, where males have higher odds of suffering HGIs.

### Overall Hip and Groin Injuries

HGIs have been associated with male athletes, since a well-known review by Orchard et al. [11] reported an overall risk ratio of 2.45 when men and women played the same sport, and this paradigm has not changed since its publication. However, emerging research has obtained results that contradict this statement. Two studies in elite women’s football showed that HGIs were amongst the most common injuries, along with the knee and ankle injuries [35, 36]. Similarly, no prevalence difference was found between the sexes in amateur football, despite the lower level of play [31]. In elite female rink hockey, HGIs account for 31% of all injuries, ranking as the second most impacted area following the thigh [37]. Wörner et al. [38] reported a 62% seasonal prevalence of HGIs in professional female ice hockey players (26% were TL), meaning that two out of three players experienced HGIs that negatively affected their performance. Overall, current evidence indicates a high occurrence of HGIs in female players, this suggests that practitioners should focus their attention on these injuries in female athletes.

### Injury definition

The sub-analyses according to TL and NTL revealed that males have the same odds of sustaining HGIs compared to females. Different results have been obtained for the injury prevalence in both sexes, varying according to the injury definition used. A study conducted on the NCAA ice hockey league over six seasons, reported that NTL injuries were more prevalent in females, while the opposite was observed for TL injuries [39]. Our results suggest that males and females displayed similar prevalence of TL injuries. It should be underscored that TL injuries represent merely the tip of the iceberg in the context of HGIs, since numerous athletes continue to participate despite experiencing symptoms, consequently leading to diminished athletic performance [6, 40, 41]. The definition of TL injuries captures only 10–30% of groin-related issues in male football and 6% in field hockey [8, 40, 41]. In the context of amateur female football, NTL injuries were found to be three times more prevalent than TL injuries [6]. It is important to point that this proportion can depend on the method used to collect injury data even when using the same tool, since results are subjected to respondent subjectivity and personal context [42]. Thus, Thorarinsdottir et al. [43] reported that 80% of groin problems caused sporting absence while Harøy et al. showed the opposite [8]. It is recommended to consider NTL injuries, as an exclusive record of TL injuries leads to an underestimation of the true impact of HGIs across both sexes [2, 6, 44]. It should be noted that the application of TL and NTL definitions and the methods used to record injuries varied across studies, including athlete-reported questionnaires [7, 32], physiotherapist reports [33], and the OSTRC [8, 34].

### Groin region

Since the 2015 Doha agreement, groin and hip pain were considered as independent categories, as groin pain (GP) was divided into four clinical entities (adductor, iliopsoas, inguinal and pubic-related GP) and hip-related pain (i.e., femoroacetabular impingement or labral tears) was considered a different source of symptoms [45]. In our study, there were no differences in injury prevalence between both sexes in the groin region. However, studies point that the prevalence does differ depending on the clinical entity affected. Iliopsoas-related GP seems to be higher in females (27%) compared to males (8–12%) [43]. Adductor-related GP is the most common affected entity in both sexes, but the prevalence is higher in males (64–68%) than in females (55%) [43]. Furthermore, the severity of adductorrelated injuries appears to be more pronounced in male players, with over 55% of injuries requiring one week of activity cessation, compared to only 30% in female players [43, 46]. Although the overall GP prevalence does not differ by sex when grouping all entities of the Doha agreement together, it is important to examine each entity individually, as the prevalence of each differs between the sexes.

### Sport

Our results indicate that odds of sustaining HGIs is identical regardless of sex in hockey players. We are confident that these results are trustworthy since heterogeneity was null and overall QoE was moderate. At an elite level, male and female ice hockey players have similar prevalence in HGIs, but substantial injuries are more prevalent in females [34]. Injury burden should also be taken into consideration when working in an ice hockey environment, since females may need longer recovery periods after suffering a groin injury due to their predisposition to suffer more severe episodes. The study by Quintana-Cepedal et al. [32] reported that males and females sustain similar number of HGIs across all playing levels in rink hockey. In football, our results indicate that males have higher odds of sustaining HGIs, with a very low QoE. Two hypotheses could explain this difference: (1) male football is characterized by match congestion periods across the calendar, leaving insufficient time between games to recover properly [8], and (2) males run at higher velocity than females [47], since many injuries occur during sprinting or accelerating [47], this could make them more prone to HGIs. It must be acknowledged that the true causes of these prevalence differences are still unknown.

### Methodological Considerations

This study is not without limitations. First, a substantial number of studies were excluded due to the lack of injury prevalence data or separate prevalence data for both sexes, resulting in only nine studies meeting the eligibility criteria. Secondly, overall certainty in the evidence appraised was low, with only two of the meta-analyses achieving moderate (hockey) or high quality (NTL). Moreover, high heterogeneity was observed across several meta-analyses. This may be explained by differences in sport type, injury definitions, level of play, and study design. The limited number of studies in some subgroups prevented sensitivity analyses. Future research should aim for more standardized definitions and homogeneous study designs to reduce heterogeneity and improve comparability. Thirdly, studies focusing solely on the groin region did not specify the affected structures (adductor, iliopsoas, inguinal, or pubic), limiting our ability to compare injury prevalence according to the Doha agreement clinical entities [45]. On this point, it is recommended for future studies to collect hip and groin injuries separately and report which entity is causing the injury [48]. Another point to note is that although the initial meta-analysis showed an OR of 1.38, the final inclusion of all eligible studies [29] resulted in the reported OR of 1.39, without altering the overall conclusions of the research. Finally, in most included studies, HGIs were recorded through self-report rather than standardized clinical assessment, which introduces a potential risk of misclassification. This may have resulted in under-reporting or inconsistent categorization of injuries, potentially obscuring true sexrelated differences. Studies should also employ consistent TL and NTL criteria and focus on sport- and sex-specific factors to better understand the mechanisms underlying observed injury patterns, as recent evidence indicates that groin injuries frequently lead to subsequent injuries, especially in the groin and hamstring muscles, highlighting the importance of targeted prevention and rehabilitation strategies [49].

On the contrary, this is the first review to perform a meta-analysis of differences in HGIs prevalence between males and females. Even though the quality of evidence was very low in four of the six sub-analyses, we calculated the prediction interval (PI), a useful measure that captures the likely effect size of a future study on the same topic [50]. The large sample of females provided better statistical power than the individual studies lacked. This review challenges the widespread belief that the prevalence of HGIs is higher in males compared to females, suggesting that female players need more attention in terms of injury monitoring and the implementation of preventive measures [51].

### Practical implications

– Males and females have similar prevalence of hip and groin injuries.
– It is necessary to implement and monitor prevention strategies for all players irrespective of their sex.
– Male football players have higher odds of sustaining hip and groin injuries compared to female football players.

## CONCLUSIONS

This systematic review and meta-analysis reveals a similar prevalence of HGIs in both males and females (OR = 1.39). This similarity persists across various injury definitions such as time-loss (OR = 1.55), non-time-loss (OR = 0.97), or injuries to the groin region (OR = 1.55), and across different sport disciplines. An exception is observed in football, where male players exhibit higher prevalence (OR = 2.25). The findings underscore the need for implementing monitoring and prevention strategies for all players, irrespective of their sex.

## Supplementary Material

Sex differences in hip and groin injury prevalence: a systematic review and meta-analysis of 3133 team-sport athletes
