# Supplementary material for: Sex differences in hip and groin injury prevalence: a systematic review and meta-analysis of 3133 team-sport athletes
Source: Biol Sport. 2026 Jan 23;43:753–64. doi: 10.5114/biolsport.2026.158668 (PMC13221697; doi:10.5114/biolsport.2026.158668)
Supplement: Sex differences in hip and groin injury prevalence: a systematic review and meta-analysis of 3133 team-sport athletes [file JBS-43-57442-s1.pdf]

SUPPLEMENTARY

APPENDIX 1. Search strategy

PUBMED/MEDLINE	
#1	((((((((((((groin pain [Title/Abstract]) OR (groin injury [Title/Abstract])) OR (adductor tendinopathy [Title/Abstract])) OR (adductor strain [Title/Abstract])) OR (Sportsman's hernia [Title/Abstract])) OR (Incipient hernia [Title/Abstract])) OR (Inguinal disruption [Title/Abstract])) OR (Core muscle injury [Title/Abstract])) OR (osteitis pubis [Title/Abstract])) OR (Pubic symphysis pathology [Title/Abstract])) OR (Rectus abdominis strain [Title/Abstract])) OR (athletic pubalgia [Title/Abstract])) OR (pubalgia [Title/Abstract])) OR (inguinal pain [Title/Abstract])
#2	((risk factor [Title/Abstract]) OR (risk marker [Title/Abstract])) OR (causal factor [Title/Abstract])
#3	#1 AND #2

APPENDIX 2. QUIPS tool item description and criteria for score (extracted from Hayden JA et al 2006).

Potential Bias	Items To Be Considered for Assessment of Potential Opportunity for Bias
**Study participation**<br>The study sample represents the population of interest on key characteristics, sufficient to limit potential bias to the results.<br>Yes<br>Partly<br>No<br>Unsure	The source population or population of interest is adequately described for key characteristics.<br>The sampling frame and recruitment are adequately described, possibly including methods to identify the sample (number and type used, e.g., referral patterns in health care), period of recruitment, and place of recruitment (setting and geographic location)<br>Inclusion and exclusion criteria are adequately described (e.g., including explicit diagnostic criteria or "zero time" description).<br>There is adequate participation in the study by eligible individuals.<br>The baseline study sample (i.e., individuals entering the study) is adequately described for key characteristics.
**Study attrition**<br>Loss to follow-up (from sample to study population) is not associated with key characteristics (i.e., the study data adequately represent the sample), sufficient to limit potential bias.<br>Yes<br>Partly<br>No<br>Unsure	Response rate (i.e., proportion of study sample completing the study and providing outcome data) is adequate.<br>Attempts to collect information on participants who dropped out of the study are described.<br>Reasons for loss to follow-up are provided.<br>Participants lost to follow-up are adequately described for key characteristics.<br>There are no important differences between key characteristics and outcomes in participants who completed the study and those who did not.
**Prognostic factor measurement**<br>The prognostic factor of interest is adequately measured in study participants to sufficiently limit potential bias.<br>Yes<br>Partly<br>No<br>Unsure	A clear definition or description of the prognostic factor measured is provided (e.g., including dose, level, duration of exposure, and clear specification of the method of measurement).<br>Continuous variables are reported or appropriate (i.e., not data-dependent) cut-points are used.<br>The prognostic factor measure and method are adequately valid and reliable to limit misclassification bias (e.g., may include relevant outside sources of information on measurement properties, also characteristics, such as blind measurement and limited reliance on recall).<br>Adequate proportion of the study sample has complete data for prognostic factors.<br>The method and setting of measurement are the same for all study participants.<br>Appropriate methods are used if imputation is used for missing prognostic factor data.
**Outcome measurement**<br>The outcome of interest is adequately measured in study participants to sufficiently limit potential bias.<br>Yes<br>Partly<br>No<br>Unsure	A clear definition of the outcome of interest is provided, including duration of follow-up and level and extent of the outcome construct.<br>The outcome measure and method used are adequately valid and reliable to limit misclassification bias (e.g., may include relevant outside sources of information on measurement properties, also characteristics, such as blind measurement and confirmation of outcome with valid and reliable test).<br>The method and setting of measurement are the same for all study participants.
**Confounding measurement and account**<br>Important potential confounders are appropriately accounted for, limiting potential bias with respect to the prognostic factor of interest.<br>Yes<br>Partly<br>No<br>Unsure	All important confounders, including treatments (key variables in conceptual model), are measured.<br>Clear definitions of the important confounders measured are provided (e.g., including dose, level, and duration of exposures).<br>Measurement of all important confounders is adequately valid and reliable (e.g., may include relevant outside sources of information on measurement properties, also characteristics, such as blind measurement and limited reliance on recall).<br>The method and setting of confounding measurement are the same for all study participants.<br>Appropriate methods are used if imputation is used for missing confounder data.<br>Important potential confounders are accounted for in the study design (e.g., matching for key variables, stratification, or initial assembly of comparable groups).<br>Important potential confounders are accounted for in the analysis (i.e., appropriate adjustment).
**Analysis**<br>The statistical analysis is appropriate for the design of the study, limiting potential for presentation of invalid results.<br>Yes<br>Partly<br>No<br>Unsure	There is sufficient presentation of data to assess the adequacy of the analysis.<br>The strategy for model building (i.e., inclusion of variables) is appropriate and is based on a conceptual framework or model.<br>The selected model is adequate for the design of the study.<br>There is no selective reporting of results.

Criteria used for scoring the QUIPS tool

To be considered as a "Low Risk of Bias (RoB) study", each study should have at least five items with low RoB. If an article had 4 items with low RoB and at least one moderate RoB item, it was considered as a "moderate RoB study". If an study has £ 4 items with low RoB, it was considered as a "high RoB study".

For each area (i.e. Study participation), "Low risk" was reached by scoring a "yes" for 75% or more of the criteria for that single category; if an area scored between 50% and 75% of "yes" and has a "partial" response, it was considered as "moderate risk". If an area had less of 75% of "yes" and any "partial" response, it was considered as "high risk".

Section and Topic	Item #	Checklist item	Location where item is reported
**TITLE**			
Title	1	Identify the report as a systematic review.	753
**ABSTRACT**			
Abstract	2	See the PRISMA 2020 for Abstracts checklist.	NA
**INTRODUCTION**			
Rationale	3	Describe the rationale for the review in the context of existing knowledge.	753
Objectives	4	Provide an explicit statement of the objective(s) or question(s) the review addresses.	753-754
**METHODS**			
Eligibility criteria	5	Specify the inclusion and exclusion criteria for the review and how studies were grouped for the syntheses.	754
Information sources	6	Specify all databases, registers, websites, organisations, reference lists and other sources searched or consulted to identify studies. Specify the date when each source was last searched or consulted.	754
Search strategy	7	Present the full search strategies for all databases, registers and websites, including any filters and limits used.	754
Selection process	8	Specify the methods used to decide whether a study met the inclusion criteria of the review, including how many reviewers screened each record and each report retrieved, whether they worked independently, and if applicable, details of automation tools used in the process.	754
Data collection process	9	Specify the methods used to collect data from reports, including how many reviewers collected data from each report, whether they worked independently, any processes for obtaining or confirming data from study investigators, and if applicable, details of automation tools used in the process.	755
Data items	10a	List and define all outcomes for which data were sought. Specify whether all results that were compatible with each outcome domain in each study were sought (e.g. for all measures, time points, analyses), and if not, the methods used to decide which results to collect.	755
	10b	List and define all other variables for which data were sought (e.g. participant and intervention characteristics, funding sources). Describe any assumptions made about any missing or unclear information.	755
Study risk of bias assessment	11	Specify the methods used to assess risk of bias in the included studies, including details of the tool(s) used, how many reviewers assessed each study and whether they worked independently, and if applicable, details of automation tools used in the process.	755
Effect measures	12	Specify for each outcome the effect measure(s) (e.g. risk ratio, mean difference) used in the synthesis or presentation of results.	755
Synthesis methods	13a	Describe the processes used to decide which studies were eligible for each synthesis (e.g. tabulating the study intervention characteristics and comparing against the planned groups for each synthesis (item #5)).	754
	13b	Describe any methods required to prepare the data for presentation or synthesis, such as handling of missing summary statistics, or data conversions.	NA
	13c	Describe any methods used to tabulate or visually display results of individual studies and syntheses.	755
	13d	Describe any methods used to synthesize results and provide a rationale for the choice(s). If meta-analysis was performed, describe the model(s), method(s) to identify the presence and extent of statistical heterogeneity, and software package(s) used.	755
	13e	Describe any methods used to explore possible causes of heterogeneity among study results (e.g. subgroup analysis, meta-regression).	755
	13f	Describe any sensitivity analyses conducted to assess robustness of the synthesized results.	NA

Section and Topic	Item #	Checklist item	Location where item is reported
Reporting bias assessment	14	Describe any methods used to assess risk of bias due to missing results in a synthesis (arising from reporting biases).	755
Certainty assessment	15	Describe any methods used to assess certainty (or confidence) in the body of evidence for an outcome.	755
**RESULTS**			
Study selection	16a	Describe the results of the search and selection process, from the number of records identified in the search to the number of studies included in the review, ideally using a flow diagram.	755
	16b	Cite studies that might appear to meet the inclusion criteria, but which were excluded, and explain why they were excluded.	755
Study characteristics	17	Cite each included study and present its characteristics.	755 and 758
Risk of bias in studies	18	Present assessments of risk of bias for each included study.	758
Results of individual studies	19	For all outcomes, present, for each study: (a) summary statistics for each group (where appropriate) and (b) an effect estimate and its precision (e.g. confidence/credible interval), ideally using structured tables or plots.	Table 1
Results of syntheses	20a	For each synthesis, briefly summarise the characteristics and risk of bias among contributing studies.	Figure 2
	20b	Present results of all statistical syntheses conducted. If meta-analysis was done, present for each the summary estimate and its precision (e.g. confidence/credible interval) and measures of statistical heterogeneity. If comparing groups, describe the direction of the effect.	758-759
	20c	Present results of all investigations of possible causes of heterogeneity among study results.	NA
	20d	Present results of all sensitivity analyses conducted to assess the robustness of the synthesized results.	759
Reporting biases	21	Present assessments of risk of bias due to missing results (arising from reporting biases) for each synthesis assessed.	NA
Certainty of evidence	22	Present assessments of certainty (or confidence) in the body of evidence for each outcome assessed.	759
**DISCUSSION**			
Discussion	23a	Provide a general interpretation of the results in the context of other evidence.	760
	23b	Discuss any limitations of the evidence included in the review.	761-762
	23c	Discuss any limitations of the review processes used.	761-762
	23d	Discuss implications of the results for practice, policy, and future research.	761-762
**OTHER INFORMATION**			
Registration and protocol	24a	Provide registration information for the review, including register name and registration number, or state that the review was not registered.	754
	24b	Indicate where the review protocol can be accessed, or state that a protocol was not prepared.	754
	24c	Describe and explain any amendments to information provided at registration or in the protocol.	NA
Support	25	Describe sources of financial or non-financial support for the review, and the role of the funders or sponsors in the review.	✓
Competing interests	26	Declare any competing interests of review authors.	✓
Availability of data, code and other materials	27	Report which of the following are publicly available and where they can be found: template data collection forms; data extracted from included studies; data used for all analyses; analytic code; any other materials used in the review.	

From: Page MJ, McKenzie JE, Bossuyt PM, Boutron I, Hoffmann TC, Mulrow CD, et al. The PRISMA 2020 statement: an updated guideline for reporting systematic reviews. BMJ 2021;372:n71. doi: 10.1136/bmj.n71.

Articles published in the Biology of Sport are licensed under an open access Creative Commons CC BY 4.0 license.
